# A case report of late-onset symptoms of erythrocytosis in univentricular dextrocardia

**DOI:** 10.1097/MD.0000000000021092

**Published:** 2020-07-02

**Authors:** Lishui Shen, Jin Zhao, Tingting Yuan, Mengjie Li, Yun Cheng

**Affiliations:** a National Center for Cardiovascular Diseases, Chinese Academy of Medical Sciences and Peking Union Medical College, Beijing; b Department of Radiology; c Department of Intensive Care Unit; d Department of Hematology; e Department of Ultrasound, Zhejiang Hospital, Hangzhou, China.

**Keywords:** dextrocardia, erythrocytosis, univentricular deformity

## Abstract

**Rationale:**

Univentricular dextrocardia is a rare congenital heart disease that usually presents cyanotic manifestations from childhood. Due to the sustained dysfunction of blood oxygenation, it is very difficult to keep an asymptomatic survival. Herein, we described an interesting case of univentricular dextrocardia who suffered from initial symptoms in his middle age.

**Patient concerns:**

A 54-year-old male patient with numbness and tingling of limbs was admitted to hospital due to the secondary manifestations of congenital heart disease.

**Diagnosis:**

The patient was diagnosed as univentricular dextrocardia with pulmonary hypertension and secondary erythrocytosis based on computed tomography (CT) scan, echocardiography, and laboratory examinations.

**Interventions:**

Intravenous hydration therapy with normal saline successfully eliminated his hyperviscosity associated symptoms. In view of socio-economic reasons, this patient refused surgical evaluation and further medical interventions.

**Outcomes:**

During 18-month follow up, he received no drug except for regular water intake. Fortunately, his life quality was satisfactory, and no other symptoms emerged except for mild numbness of limbs.

**Lessons:**

In univentricular dextrocardia, it is possible to keep a long-term asymptomatic period due to the slow progress of pathophysiology. In this population, regular cardiac function evaluation and avoiding dehydration may help improve the quality of life.

## 1 Introduction

Dextrocardia, characterized by a right-sided location of the heart within the thoracic cavity, is a rare congenital cardiac anomaly with an estimated prevalence of 1 in 10,000 to 12,000 births.^[1]^ It may occur independently or as part of situs inversus.^[2]^ Studies revealed that dextrocardia was commonly accompanied by other complex cardiac malformations such as univentricular anatomy, pulmonary atresia and tricuspid atresia.^[3]^ Dextrocardia with single ventricle (SV) indicates poor prognosis and could result in cyanosis in early stage. It is a rare condition to survive into adulthood asymptomatically without surgical intervention.^[4]^ We herein reported a case of dextrocardia with SV and late-onset symptomatic erythrocytosis. The patient has provided informed consent for these studies and their publication, and this report was approved by the Ethics committee of the Zhejiang Hospital.

## 2 Case report

A 54-year-old Asian male was admitted to hospital with the complaint of numbness and tingling of limbs for 1 month. He had no history of similar episodes in the past. On admission vital signs were as follows: temperature 37.1°C, blood pressure 112/73 mm Hg, pulse 72 beats/min, respiratory rate 18 breaths/min, finger oxygen saturation 85% (room air). Cardiac physical examination indicated dextrocardia: the apex of the heart was located in the right side of the chest accompanied with a systolic murmur at the apex. Other pertinent examinations showed cyanosis of lips, flushing of face and limbs, and a normal neurological examination.

**Figure 1:**
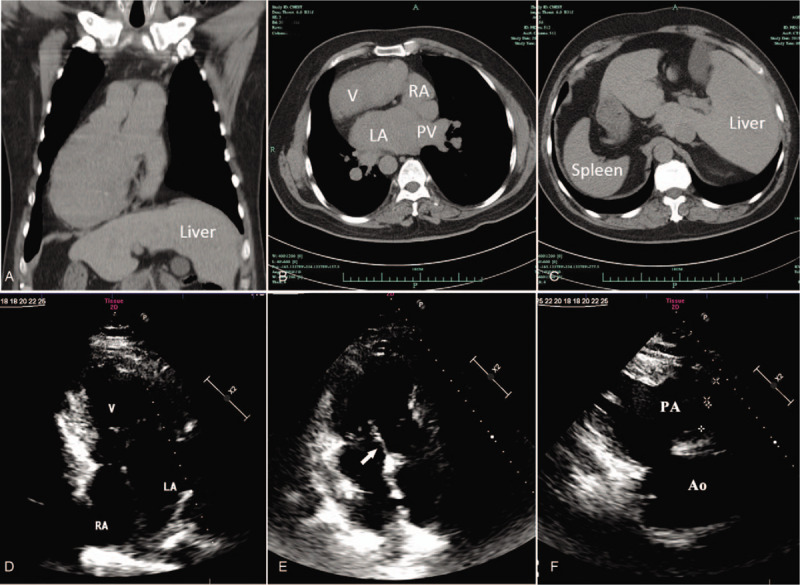
The images of computed tomography and transthoracic echocardiography of this patient. (A-C) CT showing heart in the right hemithorax with situs inversus and dilated single ventricle. (D-E) Echocardiography from apical 4-chamber view showing univentricular malformation with 2 normal sized left atrium and lengthy leaflets of tricuspid valve (white arrow). (F) Echocardiography from parasternal long-axis scan demonstrating both aorta (Ao) and pulmonary artery (PA) arises from single ventricle.

**Figure 2:**
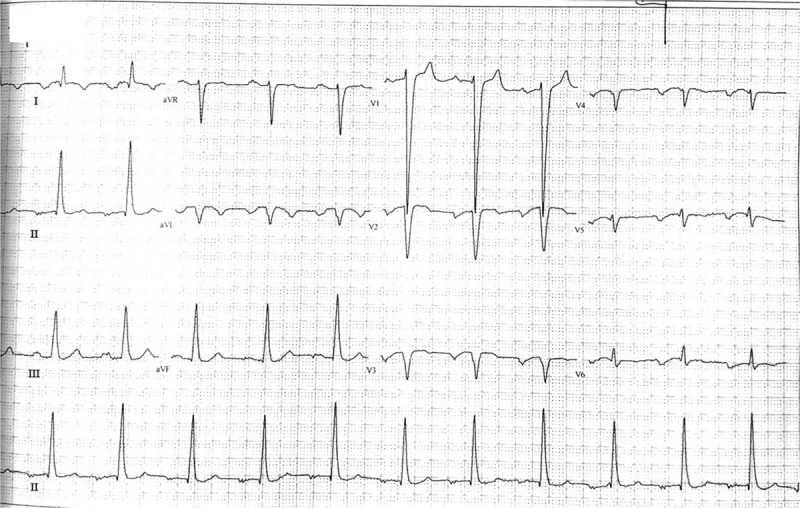
Electrocardiogram of patient with univentricular dextrocardia who presented with numbness and tingling of limbs.

The cranial magnetic resonance imaging excluded the possibility of cerebrovascular accidents. The coronal and transverse sections of chest computed tomography (CT) demonstrated the dextrocardia with situs inversus and dilated single ventricle (Fig. 1, A–C). Electrocardiography (ECG) showed inverted, broad, notched P waves in lead I and lead avL; positive P waves in avR; inverted QRS complex in leads V1 to V5 and a first-degree atrioventricular block (Fig. 2). Transthoracic echocardiography also presented the right-sided cardiac images with normal sized atriums, a double-inlet and outlet left ventricle, lengthy leaflets of tricuspid valve and mild atrioventricular valve regurgitation (Fig. 1, D-F). The pressure gradient of ventricular outflow tract to pulmonary was further estimated by pulsed doppler blood flow spectrum. The acceleration time of 85ms indicated mild-moderate pulmonary hypertension. Despite a normal coagulation function, the blood tests showed the increased hemoglobin of 19.9 g/dL and hematocrit of 58.8%, decreased oxygen partial pressure of 49.5 mm Hg and oxygen saturation of 81.3% (Table 1). The iron study indicated a mild decrease of ferritin and transferrin saturation. Other biochemical data including a blood urea nitrogen (BUN) of 7.0 mmol/L, serum creatinine of 88.3 μmol/L and creatinine clearance rate of 78.0 mL/min/1.73 m^2^ demonstrated no renal dysfunction.

**Table 1:**
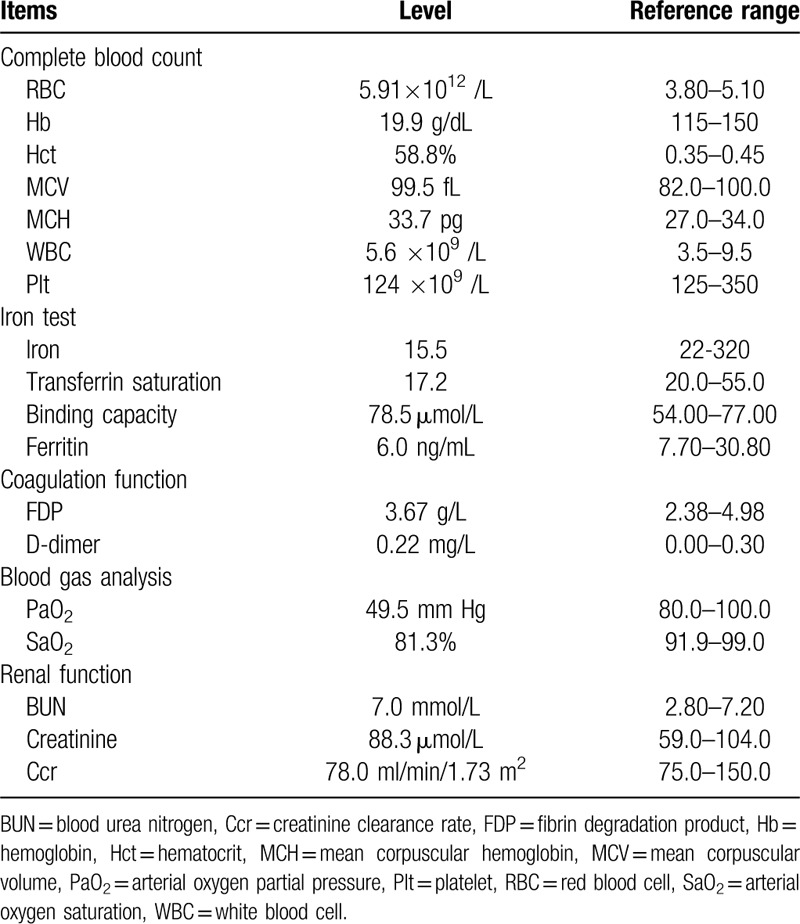
Laboratory data.

Finally, this patient was diagnosed as dextrocardia with pulmonary hypertension and secondary erythrocytosis. Hydration therapy of intravenous drip 1L normal saline within 2 hours successfully eliminated his symptoms of hyperviscosity status. In view of socio-economic reasons, the patient refused bone marrow aspiration, surgical evaluation and other further medical intervention. During 18-month follow up, he received no drug except for regular water intake. Fortunately, his life quality was satisfactory, and no other symptoms emerged except for mild numbness of limbs.

## 3 Discussion and conclusion

In this case report, we presented a rare long-term asymptomatically survived patient with dextrocardia and severe ventricular malformation. The late-onset symptoms associated with erythrocytosis promoted his medical consultation. The echo and cardiovascular imaging further revealed a double-inlet and outlet single ventricle in his heart with occult pulmonary hypertension. Univentricular dextrocardia is an extremely rare congenital heart disease which could present cyanotic manifestations in childhood due to the incomplete blood oxygenation. Currently, the prevalence of univentricular anatomy in dextrocardia has been reported to be 16% to 25%.^[2–5]^ In these patients, the progressed chronic hypoxemia could result in secondary erythrocytosis, which serves as a compensatory mechanism to increase circulating red blood cells and oxygen carrying capacity.^[6]^ However, just as a coin has 2 sides, the decompensated erythrocytosis may also add the risk of hyperviscosity which reduces blood flow and tissue perfusion with resultant symptoms such as headache, paresthesia, loss of concentration, visual disturbance, muscle weakness, and fatigue. Phornphutkul et al^[7]^ and Nadeem et al^[8]^ reported an elevated risk of cerebrovascular events and venous thrombosis due to the increased RBC mass in cyanotic congenital heart disease (CCHD).

Interestingly, this patient seemed to be the lucky man who could keep symptoms free for more than 5 decades without surgical intervention in early stage. This is in contrast to the published work by Moodie et al^[9]^ which reported only about 30% of patients with single ventricle could reach the age of 16 years. More recently, Samanek^[10]^ presented an estimated survival of 30% for the first year of life. Therefore, it is really a miracle to survive into late adulthood without any effective intervention. According to his pulmonary pressure and long-standing sedentary lifestyle, we speculated that this patient may have suffered from congestive heart failure in childhood actually due to increased pulmonary blood flow, although it was not severe. After a certain period of time, the obstructive disease of the pulmonary artery progressed, leading to a significantly increased pulmonary pressure after which his clinical condition stabilized. The patient's restriction of exercise can be thought of as a “result” of heart failure and/or hypoxemia. In addition, limited oxygen consumption may have delayed the manifestation of symptoms.

It is noteworthy that CCHD with symptomatic erythrocytosis as its first symptom appears to be rare in clinic. Recently, Shelonitda et al^[11]^ has reported a similar case with levocardia. In that case, a 21-year-old female presented a critical status of severe dehydration and erythrocytosis (hemoglobin of 25.2 g/dL, hematocrit of 75.8%). The therapy of intravenous rehydration eventually alleviated her symptoms effectively. To patients with severe erythrocytosis, the factors including dehydration and iron deficiency, may precipitate hyperviscosity symptoms. Accurate identification and early intervention could reduce the subsequent complications such as thromboembolism. In our univentricular case, although no dramatical increase of hemoglobin and hematocrit were observed, the fluid supplement was likewise effective for reducing the related symptoms. It follows that in patients with CCHD and erythrocytosis, moderate rehydration may serve as a preventive and therapeutic strategy.

In summary, univentricular dextrocardia is a rare congenital anomaly which could occasionally keep a long-term asymptomatic period due to the slow progress of pathophysiology. The secondary erythrocytosis and hyperviscosity due to chronic hypoxemia may present as its initial symptoms. In this population, regular cardiac function evaluation and avoiding dehydration may help improve the quality of life.

## Acknowledgments

We would like to thank Dr. Zhibin Bu for echo evaluation and drafting earlier version of this paper, without whom none of this work could have been done.

## Author contributions

**Conceptualization:** Lishui Shen, Jin Zhao, Tingting Yuan, Mengjie Li, Yun Cheng

**Formal analysis:** Lishui Shen, Jin Zhao, Tingting Yuan

**Writing – original draft:** Lishui Shen, Mengjie Li, Yun Cheng

**Writing – review & editing:** Lishui Shen, Mengjie Li, Yun Cheng

## References

[1] d’Udekem Y Xu MY Galati JC Predictors of survival after single-ventricle palliation: the impact of right ventricular dominance. J Am Coll Cardiol 2012;59:1178–85. 22440217 10.1016/j.jacc.2011.11.049

[2] Bohun CM Potts JE Casey BM A population-based study of cardiac malformations and outcomes associated with dextrocardia. Am J Cardiol 2007;100:305–9. 17631088 10.1016/j.amjcard.2007.02.095

[3] Garg N Agarwal BL Modi N Dextrocardia: an analysis of cardiac structures in 125 patients. Int J Cardiol 2003;88:143–55. 12714192 10.1016/s0167-5273(02)00539-9

[4] Perloff JK The cardiac malpositions. Am J Cardiol 2011;108:1352–61. 21861958 10.1016/j.amjcard.2011.06.055

[5] Evans WN Acherman RJ Collazos JC Dextrocardia: practical clinical points and comments on terminology. Pediatr Cardiol 2010;31:1–6. 19727926 10.1007/s00246-009-9516-0

[6] Reiss UM Bensimhon P Zimmerman SA Hydroxyurea therapy for management of secondary erythrocytosis in cyanotic congenital heart disease. Am J Hematol 2007;82:740–3. 17506064 10.1002/ajh.20925

[7] Phornphutkul C Rosenthal A Nadas AS Cerebrovascular accidents in infants and children with cyanotic congenital heart disease. Am J Cardiol 1973;32:329–34. 4725588 10.1016/s0002-9149(73)80142-0

[8] Nadeem O Gui J Ornstein DL Prevalence of venous thromboembolism in patients with secondary polycythemia. Clin Appl Thromb Hemost 2013;19:363–6. 23007895 10.1177/1076029612460425 PMC3831025

[9] Moodie DS Ritter DG Tajik AJ Long-term follow-up in the unoperated univentricular heart. Am J Cardiol 1984;53:1124–8. 6702691 10.1016/0002-9149(84)90648-9

[10] Samanek M Children with congenital heart disease: probability of natural survival. Pediatr Cardiol 1992;13:152–8. 1603715 10.1007/BF00793947

[11] Rose SS Shah AA Hoover DR Cyanotic congenital heart disease (CCHD) with symptomatic erythrocytosis. J Gen Intern Med 2007;22:1775–7. 17917783 10.1007/s11606-007-0356-4 PMC2219824

